# Editorial: Time, genetics, and complex disease

**DOI:** 10.3389/fgene.2022.1016049

**Published:** 2022-09-15

**Authors:** Luoying Zhang, Christoph W. Turck, Guang-Zhong Wang

**Affiliations:** ^1^ Key Laboratory of Molecular Biophysics of Ministry of Education, College of Life Science and Technology, Huazhong University of Science and Technology, Wuhan, Hubei, China; ^2^ Max Planck Institute of Psychiatry, Proteomics and Biomarkers, Munich, Germany; ^3^ CAS Key Laboratory of Computational Biology, Shanghai Institute of Nutrition and Health, University of Chinese Academy of Sciences, Chinese Academy of Sciences, Shanghai, China

**Keywords:** netherton syndrome, neurodevelopmental disorders (NDD), autism spectrum disorder (ASD), chronic kidney disease (CKD), intervertebral disc degeneration (IDD), circadian rhythms, peripheral clock, sleep deprivation

Decoding the genetic mechanisms of complex diseases is difficult since they typically involve hundreds of genes (Gibson, 2009; Hu et al., 2016). Additionally complicating the task is the fact that these genes can form complex interactions with each other (Gibson, 2009). These interactions change over time, further increasing the complexity of the entire system (Hu et al., 2016). The circadian clock is genetically based on a relatively simple regulatory system that can generate complex behaviors and impact health and disease in a dynamic manner (Takahashi, 2017; Rijo-Ferreira and Takahashi, 2019). Circadian regulation has been shown to be associated with several complex diseases including, but not limited, to neurodegenerative disorders (Nassan and Videnovic, 2022), chronic diseases (Chaix et al., 2019), and various aging-related disease (Rijo-Ferreira and Takahashi, 2019; Acosta-Rodríguez et al., 2021). Modeling the complex interactions of disease-related genes and other genes involved in the physiology of an organism from a circadian perspective is still in its early stages (Sun et al., 2020; Li et al., 2022).

This Research Topic aims to gather information gained from research concerned with advancing the understanding of complex diseases through time related mechanisms, especially those related to circadian regulation. In the following we provide a brief thematic overview of the diverse content of the Research Topic.

We first present research work on the regulation of circadian behavior and its effect on multiple layers. For the central clock, Lu et al. examined the effects of electroacupuncture on the phosphoproteome of the mammalian pacemaker, the suprachiasmatic nucleus, at different circadian times. More than 5,000 distinct phosphosites were quantified by mass spectrometry–based analysis, with many of them contributing to the phase shifts induced by electroacupuncture. In a mechanistic study, Wu et al. discovered that *PCBP1*, a circadian expressed gene, acts as a regulator of period length in human U2OS cells, possibly through enhancing the link between CRY1 and the CLOCK/BMAL1 complex. Xing et al. found that sleep deprivation reduces the expression of the core circadian clock gene NR1D1 in mice, which leads to a down-regulation of CYP7A1, the enzyme responsible for the conversion of cholesterol into bile acids. This in turn results in cholesterol accumulation. Lu et al. focused on the effect of acute sleep deprivation on the circadian transcriptome in mouse lung. By subjecting mice to sleep deprivation, thousands of genes were identified that showed an altered rhythmic expression pattern in the lung, which may directly or indirectly impact COVID-19 infection. In another approach, a video recording-based analytical method was developed by Niu et al. It can be used to monitor the daily feeding rhythm in fruit flies and enables the stable and reliable analysis of long-term feeding behavior in great detail.

Next, we included three review manuscripts discussing the linkage between complex diseases and circadian regulation. Li et al. reviewed the pathogenesis of circadian-related complex diseases by focusing on the liver, heart, skeletal muscle, blood and other peripheral organs. The paper highlights the notion that different circadian regulatory molecules or checkpoints are involved in different diseases. From the circadian and developmental disorders perspective, Lorsung et al. explored the link between autism spectrum disorder (ASD) and circadian regulation. Specifically, four circadian physiological factors that are disrupted in ASD (sleep–wake cycle, melatonin, cortisol, and serotonin) are elaborated upon in detail. Peripheral clocks are important components of the circadian clock that function even when the master clock is decoupled. Lee and Hong summarized the progress of utilizing 3D organoids to investigate the property of circadian peripheral clocks and their relevance for complex diseases.

Last, but not least, we included contributions on several other complex diseases. For Netherton syndrome, Wang et al. revealed a new frameshift mutation, which is located in the *SPINK5* gene, by utilizing whole exome sequencing. They also found that N-terminal and C-terminal mutations of the protein LEKT1 cause distinct disease phenotypes. For intervertebral disc degeneration, integrative data analysis by Jiang et al. revealed a gene co-expression network composed of long non-coding RNAs, miRNA, and protein-coding RNAs, all with functions that may be related to cytokine secretion and immune cells. For chronic kidney disease, Gao et al. found that mortality of this disease can be better predicted with the help of the nomogram model based standard deviation of the normal-to-normal (SDNN) R-R intervals and other clinical factors.

In summary, based on genetic data, an increasing number of complex diseases have been found to be closely linked to time, specifically to circadian-related regulation. The availability of public omics data and computational toolsets make the examination and statistical detection of linkages more straightforward. However, additional efforts and high-quality datasets are still required to fully understand the temporal dynamics of complex diseases. We hope the readers will find this Research Topic (and e-book) to be a good resource for exploring the linkage between complex diseases and time. Finally, we acknowledge all the experts that have contributed to this Research Topic and the reviewers’ excellent comments and valuable suggestions.
